# Determinants of arterial stiffness in COPD

**DOI:** 10.1186/1471-2466-14-1

**Published:** 2014-01-04

**Authors:** Surya P Bhatt, Adam G Cole, James Michael Wells, Hrudaya Nath, Jubal R Watts, John R Cockcroft, Mark T Dransfield

**Affiliations:** 1 Lung Health Center, Division of Pulmonary, Allergy and Critical Care Medicine, University of Alabama at Birmingham, Birmingham, AL, 35294, UK; 2 Department of Radiology, University of Alabama at Birmingham, Birmingham, AL, UK; 3 Department of Cardiology, Wales Heart Research Institute, University Hospital, Cardiff, UK; 4 The Birmingham VA Medical Center, Birmingham, AL, UK

**Keywords:** COPD, Arterial stiffness, Arterial calcification, Cardiovascular

## Abstract

**Background:**

Cardiovascular morbidity and mortality is high in patients with chronic obstructive pulmonary disease (COPD) and arterial stiffness is a potentially modifiable risk factor with added predictive value beyond that obtained from traditional risk factors. Arterial stiffness has been the target of pharmacologic and exercise interventions in patients with COPD, but the effects appear limited to those patients with more significant elevations in arterial stiffness. We aimed to identify predictors of increased arterial stiffness in a cohort with moderate to severe COPD.

**Methods:**

Aortic pulse wave velocity (aPWV) was measured in subjects with moderate to severe COPD enrolled in a multicenter randomized controlled trial. Subjects were categorized into quartiles based on aPWV values and factors affecting high arterial stiffness were assessed. Multivariate models were created to identify independent predictors of high aPWV, and cardiovascular disease (CVD).

**Results:**

153 patients were included. Mean age was 63.2 (SD 8.2) years and mean FEV_1_ was 55.4 (SD 15.2) % predicted. Compared to the quartile with the lowest aPWV, subjects in the highest quartile were older, had higher systolic blood pressure (SBP), were more likely to be current smokers, and had greater burden of thoracic aortic calcification. On multivariate analyses, age (adjusted OR 1.14, 95%CI 1.05 to 1.25, p = 0.003) and SBP (adjusted OR 1.06, 95% CI 1.02 to 1.09, p = 0.001) were independent predictors of elevated aPWV. Body mass index, therapy with cholesterol lowering medications and coronary calcification were independent predictors of CVD.

**Conclusions:**

Elevated arterial stiffness in patients with COPD can be predicted using age, blood pressure and thoracic aortic calcification. This will help identify subjects for enrollment in clinical trials using aPWV for assessing the impact of COPD therapies on CV outcomes.

**Trial registration:**

Clinicaltrials.gov NCT00857766

## Background

Cardiovascular morbidity and mortality is high in patients with chronic obstructive pulmonary disease (COPD) and this appears to be independent of established risks such as smoking, gender and age [1-3]. A plausible mechanistic connection is the presence of chronic inflammation in both the lung and cardiovascular system [1,4,5] which may be associated with endothelial dysfunction, [4,6] loss of elastin, [5,7,8] and eventual vascular calcification [9]. Though no disease specific marker of cardiovascular risk has been identified, many markers of this potentially shared pathophysiologic process have been demonstrated to be present in patients with COPD including elevated c-reactive protein, [1,2] arterial stiffness, [5,7,10] and thoracic aortic calcification (TAC) [9] as well as impaired flow mediated vasodilation [6]. These associations are independent of smoking status, inversely related to lung function and directly related to the extent of emphysema as detected by quantitative CT scanning [1,3,5,9].

Arterial stiffness is a potentially modifiable risk factor and has added predictive value beyond that obtained from traditional risk factors [11-13]. Arterial stiffness has been the target of pharmacologic and exercise interventions in patients with COPD [14-16]. Though these studies suggest a potential benefit with inhaled treatments and exercise, the effects with pharmacologic intervention appear limited to those patients with more significant elevations in arterial stiffness [14]. Identifying those subjects who are more likely to respond to interventions is of potential value in phenotyping and in targeting therapy. We aimed to identify predictors of increased arterial stiffness in a cohort with moderate to severe COPD.

## Methods

### Study participants

Subjects with moderate to severe COPD enrolled in a multicenter, randomized controlled study (NCT00857766) were included. Written informed consent was obtained from all participants, and the study was approved by the Institutional Review Board at the University of Alabama (IRB No. W110208001). Details of the study design have been published previously [14]. Briefly, we included patients age ≥ 50 years and with at least 10 pack years smoking history with moderate to severe COPD as evidenced by a post-albuterol forced expiratory volume in the first second (FEV_1_)/forced vital capacity (FVC) ratio ≤ 0.70, and FEV_1_ <80% predicted. Patients had to be on stable doses of medications for diabetes mellitus, hyperlipidemia and cardiovascular disease for at least 3 months prior to enrolment. Baseline demographic and clinical characteristics were used for all analyses. Cardiovascular disease included a history of coronary artery disease (angina, myocardial infarction, and ischemic heart disease without cardiomyopathy), cerebrovascular disease (stroke and transient ischemic attacks), or peripheral arterial disease.

### Blood, vascular and CT measurements

Serum was obtained for measurement of lipids, blood glucose, and inflammatory markers (highly sensitive C-reactive protein, hs-CRP; and serum fibrinogen).

Aortic pulse wave velocity (aPWV) was measured using the SphygmoCor™ system (AtCorMedical, Sydney, Australia) by sequentially recording ECG-gated carotid and femoral artery waveforms by applanation tonometry. The distance between the site of measurement over the carotid and femoral arteries was divided by the wave transit time, averaged over ten cardiac cycles.

Multidetector computed tomography (CT) was used for quantitative estimation of emphysema. Scans were obtained at full inspiration (120 kVp, 100 mAs, slice thickness 1 or 1.25-mm for 16-slice scanners and 0.5 or 0.65 mm for 64 slice scanners). Percentage emphysema was calculated using the percentage of lung volume at total lung capacity with attenuation less than −950 Hounsfield Units (HU) using the Pulmonary Workstation 2.0 Software (VIDA Diagnostics, Coralville, IA, USA). Coronary artery calcification (CAC) and thoracic aortic calcification (TAC) were assessed using a post-imaging processing application (Aquarius Work Station, TeraRecon, San Mateo, CA) using the Food and Drug Administration-approved Calcium Analysis Tool. Coronary arterial calcification score was assessed by obtaining the sum of each CT slice’s area of calcification multiplied by a coefficient based on the peak CT number (Agatston score). Areas of calcification were defined by the standard scoring threshold (*>* 130 HU). Coronary arterial calcification was first scored using the semi-automated calcium analysis software and then manually corrected by a technologist when mischaracterized by the software algorithm. A radiologist (HN or JW) then individually reviewed each case and additional corrections were made as needed. Thoracic aortic calcifications were scored separately using the same technique. Thoracic aorta was defined as the aorta, excluding brachiocephalic arteries, extending from aortic valve to the origin of the celiac axis.

### Statistical analyses

Baseline data are expressed as means with standard deviations for normally distributed values. Bivariate analyses were conducted with chi-square test for categorical data and Student t test for continuous data where appropriate. CAC and TAC were log transformed as CAC + 1 and TAC + 1. Associations with aPWV and clinical predictors were assessed using univariate and multivariate linear regression analyses. Subjects were then categorized into quartiles based on aPWV values, and comparisons were made between these groups using Analyses of Variance (ANOVA) and chi square tests as appropriate. Variables significant on univariate analyses were entered into a multinomial regression model with the lowest aPWV quartile as reference to find independent predictors of the highest aPWV. Subjects with and without CVD were compared, and predictors of CVD were assessed using logistic regression by including variables significant on univariate analyses. All analyses were performed with SPSS software (version 20.0) and a p value <0.05 was used to define statistical significance.

## Results

**Figure 1:**
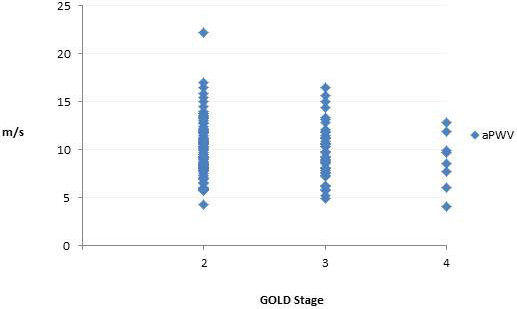
Distribution of aortic pulse wave velocity (aPWV) by GOLD stage.

Of the 249 patients enrolled in the clinical trial, 153 patients with complete CT data were included in the current analysis (Table 1). Mean age was 63.2 (SD 8.2) years. Patients were predominantly Caucasian (92%) and there was an almost equal sex distribution (54% males). Approximately half of the patients were active smokers. Of the 153 patients, 101 had moderate COPD as defined by the Global Initiative for Chronic Obstructive Lung Disease (GOLD COPD stage II) and 52 had severe COPD (GOLD stage III-IV) [17]. Patients with GOLD stage III/IV had a lower body mass index than those with GOLD stage II and as expected, also had greater % emphysema on CT. There was a modest correlation between pulse pressure and aPWV (Pearson’s r = 0.36, p < 0.01). Figure 1 shows the distribution of aPWV by GOLD COPD stage.

**Table 1:** Demographics of patients with COPD

	**All subjects (n=153)**	**GOLD II (n=101)**	**GOLD III/IV (n=52)**	**p value**
Age (years)	63.2 (8.2)	63.4 (8.7)	63.1 (7.2)	0.86
Male, n (%)	83 (55%)	56 (56%)	27 (52%)	0.63
Race, Caucasian, n (%)	140 (92%)	92 (91%)	49 (94%)	0.67
BMI (kg/m^2^)	26.9 (4.8)	27.6 (4.7)	25.5 (4.7)	0.01
Current smoker, n (%)	77 (49%)	50 (50%)	25 (48%)	0.82
Total pack-years	56.5 (29.4)	56.2 (27)	57.1 (33.8)	0.87
FEV_1_ (% predicted)	55.4 (15.2)	64.8 (7.5)	37.4 (8.4)	<0.001
FEV_1_ (L)	1.62 (0.6)	1.9 (0.52)	1.08 (0.29)	<0.001
FEV_1_/FVC	0.55 (0.11)	0.59 (0.08)	0.53 (0.09)	<0.001
Coronary artery disease, n (%)	46 (30%)	32 (32%)	14 (27%)	0.52
Diabetes mellitus,n (%)	45 (30%)	30 (30%)	15 (29%)	0.88
Cerebrovascular disease, n (%)	6 (4%)	5 (5%)	1 (2%)	0.67
Peripheral vascular disease, n (%)	13 (9%)	11 (11%)	2 (4%)	0.22
Cardiovascular disease, n (%)	53 (35%)	39 (39%)	14 (27%)	0.16
Systolic BP	127.6 (17.1)	128.8 (17)	125.3 (17.3)	0.24
Temperature (°C)	36.5 (0.3)	36.5 (0.3)	36.5 (0.4)	0.50
TAC	3556.8 (6156.9)	3923.2 (7919.4)	2852.0 (3616.1)	0.31
CAC	741.2 (1061.4)	789.6 (1094.9)	648.1 (997.3)	0.44
aPWV (m/s)	9.98 (2.85)	10.14 (2.88)	9.69 (2.79)	0.37
% CT emphysema	8.93 (11.39)	5.63 (7.32)	15.28 (14.76)	<0.001
Total cholesterol (mmol/L)	4.81 (1.1)	4.73 (1.12)	4.94 (1.04)	0.24
LDL cholesterol (mmol/L)	2.59 (0.97)	2.53 (0.92)	2.71 (1.07)	0.28
HDL cholesterol (mmol/L)	1.45 (0.53)	1.38 (0.49)	1.59 (0.58)	0.03
hsCRP (mg/L)	4.59 (7.0)	5.05 (8.16)	3.70 (3.73)	0.27
Plasma glucose (mmol/L)	5.85 (1.4)	6.05 (1.53)	5.44 (0.95)	0.01

All values expressed as mean (standard deviation) unless otherwise specified.

P value for comparisons between GOLD II and GOLD III/IV.

Definition of abbreviations: GOLD = Global initiative for Obstructive Lung Disease; BMI = body mass index; FEV_1_ = forced expiratory volume in the first 1 second; FVC = forced vital capacity; TAC = thoracic aortic calcification; CAC = coronary arterial calcification; aPWV = aortic pulse wave velocity; CT = computed tomography; LDL = low density lipoprotein; HDL = high density lipoprotein; hsCRP = high sensitivity C-reactive protein.

Univariate analysis revealed significant associations between aPWV and multiple risk factors such as age (unstandardized beta co-efficient, ß = 0.13, 95% CI 0.08 to 0.18; p < 0.001), number of pack-years of smoking (ß = 0.02, 95% CI 0.003 to 0.03; p = 0.02), systolic blood pressure (ß = 0.06, 95% CI 0.03 to 0.08; p < 0.001), temperature (ß = 1.4, 95% CI 0.06 to 2.75; p = 0.04), plasma glucose (ß = 0.37, 95% CI 0.05 to 0.69; p = 0.02), logCAC (ß = 0.19, 95% CI 0.02 to 0.36; p = 0.03), logTAC(ß = 0.41, 95% CI 0.2 to 0.62; p < 0.001), and CVD ((ß = 1.05, 95% CI 0.11 to 1.99; p = 0.03). LDL-cholesterol (ß = −0.16,95% CI = −0.63 to 0.31;p = 0.51) and treatment with lipid lowering medications (ß = 0.87,95% CI = −0.04 to 1.19;p = 0.06) were not associated with aPWV. There was no association between aPWV and markers of inflammation, C-reactive protein (ß = 0.06, 95% CI −0.01 to 0.12;p = 0.08) and fibrinogen (ß = −0.01, 95% CI −0.63 to 0.61;p = 0.96), or with diabetes mellitus (ß = 0.93,95% CI = −0.05 to 1.91;p = 0.06). There was also no association between %emphysema on CT scan and aPWV (ß = 0.03,95% CI −0.01 to 0.07;p = 0.17) though a relationship was observed when log %emphysema was used (ß = 0.33,95% CI 0.01 to 0.64;p = 0.04). On multivariate analyses, there was a significant association between aPWV and age, glucose, temperature and systolic blood pressure (Table 2).

**Table 2:** Multivariate associations between clinical variables and aortic pulse wave velocity

	**Regression co-efficient beta (95% confidence interval)**	**p value**
Age	0.1 (0.04 – 0.17)	0.001
Systolic blood pressure (mmHg)	0.04 (0.02 – 0.07)	0.001
Temperature (°C)	1.17 (0. 534 – 3.02)	0.006
Plasma glucose (mmol/L)	0.47 (0.15 – 0.78)	0.004
Pack-years	0.012 (−0.002 – 0.027)	0.09
Cardiovascular disease	0.90 (−0.029 – 1.829)	0.06
logTAC	0.18 (−0.12 – 0.47)	0.24
logCAC	−0.23 (−0.50 – 0.03)	0.08

Definition of abbreviations*:TAC* Thoracic Aortic Calcification. *CAC* Coronary Artery Calcification.

Adjusted R^2^ = 0.29.

Subjects were divided into four quartiles based on percentile aPWV (Table 3). On comparison with the quartile with the lowest aPWV, subjects in the highest quartile were older (68.1 + 8.4 vs. 59.4 + 7.2 years, p < 0.01), had higher systolic blood pressure (134.7 + 16.8 vs. 120.5 + 17.3 mmHg, p < 0.01), and had greater burden of thoracic aortic calcification (logTAC 7.64 + 1.31 vs. 6.28 + 2.16, p < 0.01) (Table 3). They were also more likely to be current smokers (68% vs. 34%, p < 0.05). Subjects in the highest quartile had a greater frequency of CVD, but this was not entered in the multivariate model as this was an outcome of interest (Table 3). On multivariate analyses, subjects in the highest aPWV quartile were older (adjusted odds ratio, OR 1.14, 95% CI 1.05 to 1.25, p = 0.003), and had greater systolic blood pressure (adjusted OR 1.06, 95% CI 1.02 to 1.09, p = 0.001). LogTAC did not reach statistical significance (adjusted OR = 1.14, 95% CI 0.82 to 1.58, p = 0.44).

**Table 3:** Comparison of variables across aPWV quartiles

	**Quartile 1 (4.1 – 8.04 m/s) n = 38**	**Quartile 2 (8.05 – 9.69 m/s) n = 36**	**Quartile 3 (9.7 – 11.74 m/s) n = 41**	**Quartile 4 (>11.75 m/s) n = 38**
Age (years) ¥	59.4 (7.2)	61.7 (6.9)	63.6 (7.7)*	68.1 (8.4)**
Male, n (%)	20 (53)	17 (47)	22 (54)	24 (63)
Race, Caucasian, n (%)	34 (89)	33 (92)	39 (95)	36 (95)
BMI (kg/m^2^)	26.4 (4.8)	27.2 (4.9)	26.1 (4.6)	28.0 (4.7)
Current smoker, n (%)	13 (34)	18 (50)	20 (49)	26 (68)*
Total pack-years	55.32 (29.81)	48.75 (23.67)	55.56 (20.59)	66.92 (38.69)
FEV_1_ (%predicted)	52.99 (15.17)	59.56 (13.43)	55.18 (15.36)	54.26 (15.97)
FEV_1_ (L)	1.61 (0.62)	1.76 (0.62)	1.60 (0.58)	1.54 (0.57)
Cardiovascular disease, n (%)	10 (26)	8 (22)	14 (34)	21 (55)*
Diabetes mellitus, n (%)	8 (21)	11 (31)	10 (24)	17 (45)
Heart Rate (per minute)	69.6 (10.3)	69.1 (11.1)	68.5 (13.4)	71.2 (8.8)
Systolic blood pressure (mmHg) ¥	120.5 (17.3)	125.9 (14.1)	128.8 (17.2)*	134.7 (16.8)**
Diastolic blood pressure (mmHg)	75.9 (10.1)	78.1 (11.1)	75.7 (8.3)	78.6 (9.3)
Log TAC	6.28 (2.16)	6.17 (2.17)	7.20 (1.97)*	7.64 (1.31)**
Log CAC	5.43 (1.80)	5.10 (2.44)	5.71 (1.97)	5.66 (2.05)
aPWV (m/s)	6.7 (1.1)	8.8 (0.4)	10.6 (0.5)	13.7 (2.1)
%emphysema on CT	7.2 (9.1)	6.6 (9.3)	11.1 (14.1)	10.3 (11.7)
LDL cholesterol (mmol/L)	2.56 (1.04)	2.66 (0.98)	2.61 (0.93)	2.60 (1.03)
HDL cholesterol (mmol/L)	1.42 (0.53)	1.51 (0.68)	1.52 (0.47)	1.35 (0.41)
Total cholesterol (mmol/L)	4.75 (0.97)	4.86 (1.10)	4.93 (1.18)	4.70 (1.16)
Log hsCRP (mg/L)	0.95 (0.88)	0.76 (0.95)	1.08 (1.02)	1.28 (1.12)
Log fibrinogen (mg/dl)	1.10 (0.21)	0.98 (0.28)*	1.07 (0.29)	1.08 (0.20)

*p<0.05. ** p<0.01 for quartiles compared with quartile 1.

¥ p<0.01 on multivariate analyses for quartile 4 compared with quartile 1.

All values expressed as mean (standard deviation) unless otherwise specified.

Definition of abbreviations: *BMI* body mass index, *FEV*_1_ forced expiratory volume in the first 1 second, *TAC* thoracic aortic calcification, *CAC* coronary arterial calcification, *aPWV* aortic pulse wave velocity, *CT* computed tomography, *LDL* low density lipoprotein, *HDL* high density lipoprotein, *hsCRP* high sensitivity C-reactive protein.

We also assessed predictors of prevalent CVD and found that BMI, markers of dyslipidemia, cholesterol lowering medications, coronary calcification and aPWV were significantly associated with CVD on univariate analyses (Table 4). On multivariate analyses, BMI, cholesterol lowering medications and logCAC were independent predictors of CVD. aPWV was not independently associated with CVD on multivariate testing (p = 0.06) (Table 4).

**Table 4:** Unadjusted and adjusted odds ratios for predictors of cardiovascular disease

	**Unadjusted odds ratio (95% confidence interval)**	**Adjusted odds ratio (95% confidence interval)**
Body mass index (kg/m^2^)	1.11 (1.03 – 1.19)**	1.12 (1.01 – 1.21)*
High density lipoprotein (mmol/L)	0.32 (0.14 – 0.72)**	-
Low density lipoprotein (mmol/L)	0.53 (0.36 – 0.78)**	-
Total cholesterol (mmol/L)	0.53 (0.37 – 0.75)**	-
logCAC	1.43 (1.16 – 1.76)**	1.40 (1.11 – 1.76)**
Cholesterol lowering medications	0.30 (0.15 – 0.61)**	0.30 (0.12 – 0.71)**
aPWV (m/s)	3.46 (1.32 – 9.07)*	3.02 (0.94 – 9.77)

*p<0.05. ** p<0.01.

CAC = Coronary artery calcification. aPWV = Aortic pulse wave velocity.

## Discussion

We have shown that in patients with moderate to severe COPD, arterial stiffness can be predicted by older age, higher systolic blood pressure and greater thoracic aortic calcification. These factors could be used to select patients for clinical trials aimed at modifying aPWV as a surrogate for CVD risk.

A large proportion of patients with COPD die from cardiovascular causes rather than from respiratory failure, especially in mild to moderate disease, and this appears to be independent of traditional risk factors for CVD [18]. Efforts to uncover novel prognostic markers have led to the identification of coronary and aortic calcification, and arterial stiffness as predictors of incident coronary artery disease [13,19]. These markers are indicative of vascular and target organ damage and add to the predictive utility of traditional risks [20-22]. Patients in the current study had a significant burden of traditional cardiovascular risk factors. We confirmed that despite this, CAC and aPWV proved to be robust predictors of CVD in COPD. This has been demonstrated in other special populations including, the very elderly and in those with other systemic and inflammatory diseases [12,23,24]. There was an inverse relationship between dyslipidemia and CVD, an effect likely explained by treatment as subjects with CVD were more likely to be on statins and cholesterol lowering medications than those without CVD (62% vs. 33%, p < 0.001). The intake of cholesterol lowering medications was independently associated with CVD.

While arterial calcification is a robust predictor of CVD, it is probably not modifiable. aPWV is the gold standard for measuring arterial stiffness and population based studies have shown that it is an independent risk factor for cardiovascular disease [25-27]. Multiple studies in the general population have shown that arterial stiffness can be modified by intervention [28,29]. Because aPWV is a dynamic measurement and is possibly a precursor to CVD, there has also been interest in the use of aPWV as a surrogate endpoint in clinical trials examining the effects of COPD therapies on cardiovascular morbidity and mortality [14,15]. Vivodtzev et al. showed that a 4 week endurance training program resulted in significant improvement in arterial stiffness in subjects with COPD [16]. This was confirmed by Gale et al. who found that pulmonary rehabilitation positively impacts arterial stiffness [30]. In both these studies, the major effector of this reduction appeared to be a reduction in blood pressure. On the other hand, we and others have shown previously that inhaled corticosteroid/long acting beta agonist likely reduces arterial stiffness, and this reduction is greatest in those with the highest stiffness [14,15]. Whether this is due to reduction in inflammation is unclear. Sabit et al. found a positive correlation between serum interleukin-6 and arterial stiffness in COPD, [10] but in another study there was no relation between C-reactive protein and arterial stiffness [5]. In the current study we also found no significant associations between markers of systemic inflammation and arterial stiffness. Beta agonists may also reduce arterial stiffness via nitric oxide synthesis and vasodilation [31]. In this study, in addition to age and blood pressure, we showed that thoracic arterial calcification can also be used to predict arterial stiffness. Interestingly, there was no association between severity of emphysema and arterial stiffness, unlike seen in a previous study [5]. It is not clear if this variance in COPD related arterial stiffness is due to differences in emphysema severity or involvement of other mechanisms that were not assessed. We did find a significant relationship between log CT emphysema and aPWV suggesting that the non-normal distribution of emphysema in this population may in part explain the lack of linear correlation.

This study has some limitations. CT scans included in this study were not EKG-gated; however, a recent study showed that non-gated computed tomography is reliable for estimation of TAC and CAC [32]. In addition, the study only included patients with COPD, precluding comparisons of strengths of association in matched controls. However, we did stratify arterial stiffness to determine contributing factors.

## Conclusions

In summary, this study demonstrates that in patients with COPD elevated arterial stiffness can be predicted using age, blood pressure and thoracic aortic calcification. This will help identify subjects for enrollment in clinical trials using aPWV for assessing the impact of COPD therapies on CV outcomes. In addition, the findings suggest that aPWV may be an important predictor of CVD in subjects with COPD.

## Competing interests

S P B, A G C, J M W, H N, and J R W report no conflicts of interest. J R C received a research grant from GlaxoSmithKline to do this study. M T D reports consultant fees and speaker fees from GlaxoSmithKline and Boerhinger Ingelheim. The authors declare that they have no competing interests.

## Pre-publication history

The pre-publication history for this paper can be accessed here:

http://www.biomedcentral.com/1471-2466/14/1/prepub
